# Efficacy and safety of the syrup “KalobaTUSS®” as a treatment for cough in children: a randomized, double blind, placebo-controlled clinical trial

**DOI:** 10.1186/s12887-020-02490-2

**Published:** 2021-01-11

**Authors:** Ilaria Carnevali, Rita La Paglia, Lara Pauletto, Floriana Raso, Marco Testa, Carmen Mannucci, Emanuela Elisa Sorbara, Gioacchino Calapai

**Affiliations:** 1 Scientific Affairs Department Schwabe Pharma Italia, Egna, BZ Italy; 2 Pediatrician, Azienda Sanitaria Provinciale 5, Messina, Italy; 3 Department of Biomedical, Dental, Morphological and Functional Imaging Sciences, University of Messina, Via Consolare Valeria n 1, 98125 Messina, Italy

**Keywords:** Syrup, Cough, Children, Honey, *Malva sylvestris*, *Inula helenium*, *Plantago major*, *Helichrysum stoechas*

## Abstract

**Background:**

Acute cough in children often causes discomfort to children and parents, reducing their quality of life. Despite the extensive utilization of over-the-counter remedies for cough, the efficacy of most of these treatments in children has not been confirmed.

**Methods:**

We conducted a randomized, double blind, placebo-controlled clinical trial of 106 children with acute cough to evaluate the efficacy and safety of KalobaTUSS**®**, a paediatric cough syrup based on acacia honey and on *Malva sylvestris* extract, *Inula helenium* extract, *Plantago major* extract, and *Helichrysum stoechas* extract by using a validated 6 points Likert scale.

**Results:**

Children were orally treated with KalobaTUSS**®** or placebo for 8 days. Children receiving KalobaTUSS**®** showed an early and significant reduction in night-time and day-time cough scores measured using a specific scale and a shorter duration of cough than children treated with the placebo.

**Conclusions:**

KalobaTUSS**®** is well tolerated and produces positive effects by reducing the severity and shortening the duration of cough in children.

**Trial registration:**

Clinicaltrials.gov no. NCT04073251. Retrospectively registered.

## Background

Cough is classified as acute or chronic. Acute cough is a very common symptom resolving in the short term, while chronic cough lasts for more than 3 weeks [1]. Acute cough in children is a common reason why parents seek medical treatment and it is a real challenge for paediatricians. Cough is probably the most bothersome symptom associated with upper respiratory tract infections for children and their parents. Cough frequently produces distress and sleep disorders in the whole family [2].

In most cases, acute cough is self-restrained, but its perseverance can be exasperating and can worsen the quality of life and common social participation [3].

The effect of cough experienced by children on the family’s life may create increasing discomfort. Indeed, children with acute cough may experience a transient disability, prompting parents to miss work and children to miss school, thus increasing the global community cost [4]. Generally, parents’ concerns increase when their children’s cough lasts for more than a week. Consequently, parents often seek for a medical consultation with a requirement for drug treatment, although most antitussive drugs lack evidence of effectiveness [5].

In recent years, the inappropriate prescription of antitussive pharmacological treatments in children has decreased; however, paediatricians’ prescriptions currently do not always reflect an accurate treatment of cough [3]. According to epidemiological data, cough occurring in children produces more anxiety than in cough occurring in adults, and many people view this symptom as “a disease”. A recent Italian survey confirms this view, suggesting that some inclinations of paediatricians’ therapeutic practice should be modified to achieve better control of cough in children and to reduce its impact [3]. Moreover, when attempting to treat cough, children are administered over-the-counter products with little or no scientific evidence of proven efficacy [6].

The World Health Organization has identified honey as a possible demulcent treatment for cough [7, 8]. Demulcent is a substance that is usually based on polysaccharides, covers the throat and reduces pain when the mucosa is irritated by increasing saliva production to reduce the cough reflex [9].

Parents often believe that the administration of honey to relieve cough and improve sleep quality at night is more desirable than the administration of drugs such as diphenhydramine or dextromethorphan. Moreover, evidence from some clinical studies support the use of honey to relieve cough. Results from a study comparing a single dose of honey, dextromethorphan and no treatment indicated that parents view honey as the most favourable treatment for symptomatic relief of nocturnal cough due to upper respiratory tract infection in children aged 2–18 years [10].

Another study in which the effects of honey on the nightly cough and sleep quality of children and their parents were compared with dextromethorphan and diphenhydramine showed that the administration of honey before sleep produced a greater effect on alleviating cough induced by an upper respiratory infection in children aged 24–60 months [11].

In 2018, the Cochrane Collaboration reviewed six randomized controlled clinical studies that investigated the use of honey to treat acute cough in children. The authors concluded that honey alleviates cough with a greater size effect than diphenhydramine, an antihistamine drug classified as a cough suppressant in the United States, placebo or no treatment and with little or no difference compared to dextromethorphan [12].

Due to assumptions described above, an evaluation of a different approach to treat cough, instead of synthetic drugs, appears to be interesting. The safeguarding effect of a mechanical barrier in the throat may be considered a well-founded therapeutic approach that indirectly exhibits anti-inflammatory activity and is able to reduce the damage produced by irritant agents or microbes. This protective activity may be acquired from natural substances such as honey and plant extracts.

Based on the considerations listed above and the lack of effective paediatric antitussive products, a cough syrup for children (KalobaTUSS®) based on acacia honey in combination with *Malva sylvestris*, *Inula helenium*, *Plantago major* and *Helichrysum stoechas* extracts has been developed for acute cough in children. This syrup is a Medical Device class IIa, classified in accordance with Directive 93/42/EC.

*Malva sylvestris* is a biennial–perennial herbaceous medicinal plant known as “common mallow” that is indigenous to North Africa, Asia and Europe. It has been used in folk medicine for its mucus formed of flavonoids and polysaccharides. Traditionally, *M. sylvestris* has been used to treat upper respiratory tract infections. The entire plant possesses beneficial properties, and it has been used in folk medicine for its mucus because the leaves and flowers are rich in flavonoids and mucilage. Mucilage is present at a percentage of 6–8% in *M. sylvestris* leaves and is composed of high-molecular weight acidic polysaccharides of the rhamnogalacturonan type that are also observed in epidermal cells [13]. In 2018, the European Medicinal Agency (EMA) assessed the traditional use of *M. sylvestris* as a “demulcent preparation for the symptomatic treatment of oral or pharyngeal irritation and associated dry cough” [14, 15]. The extract used in the syrup is a *Malva sylvestris* leaf aqueous extract, containing no less than 80% mucilage.

*Inula helenium* (Elecampane) is a perennial herbaceous plant of the Asteraceae family that is native to England and Europe, but it also grows in the northern and eastern United States, Canada Asia, India and Siberia. This plant was used to treat cough based on its medicinal properties [16].

*Inula helenium* roots are rich in coumarins, flavonoids, polysaccharides (up to 44% inulin and pectic substances), fatty acids and saponins [17]. The sesquiterpene lactones present in the phytocomplex are responsible for the expectorant properties of this plant [18]. Traditionally, extracts of the plant have been used to treat bronchial/tracheal catarrh and dry irritating cough in children [19]. The extract used in the syrup is an *Inula helenium* roots aqueous extract, containing no less than 30% polysaccharides.

*Plantago major* is a plant of the Plantaginaceae family that is native to temperate areas of Asia and Europe. Aerial parts contain mucilage (up to 12%). In traditional medicine, the plant was employed as a treatment for cough related to inflammation of the upper respiratory airways [20]. The plant is a quite effective soothing, moistening, expectorant for a dry irritable cough, because the mucous membranes are unable to produce the immune factor-rich mucous that coats, soothes and protects the membrane; thus, it becomes dry, inflamed and easily irritated [21]. Plants belonging to the genus *Plantago* significantly attenuate cough [22, 23]. The extract used in the syrup is a *Plantago major* aerial part aqueous extract, containing no less than 30% polysaccharides.

*Helichrysum stoechas* is an annual herb belonging to the family Asteraceae and is indigenous to the occidental Mediterranean regions [24]. The extract used in the syrup is a *Helichrysum stoechas* aerial part aqueous extract, containing no less than 30% polysaccharides.

We performed a randomized, controlled double blind clinical trial to investigate the effects of KalobaTUSS**®**, an innovative syrup containing acacia honey and herbal extracts of *Malva sylvestris*, *Inula helenium*, *Plantago major* and *Helichrysum stoechas,* on acute cough in children aging 3–6 years. The extract of *Malva sylvestris* is titrated to 80% based on mucilage, and the extracts of *Inula*, *Plantago* and *Helichrysum* are titrated to 30% based on polysaccharides. The rich composition of the study syrup makes it suitable for use as a treatment for cough in children, because it forms a mechanical barrier on the mucosa. Mucilage is a complex of polysaccharide molecules that is part of different organs of the plant, mainly the aerial parts. When in contact with water, mucilage tends to swell and to create a jelly-like film on the contact surface. When mucilage contacts the respiratory mucosa, it adsorbs moisture present in the mucus, gels and forms bonds with the structure of the mucosa [25]. In the presence of an inflammatory state, this film exerts a soothing and emollient effect on irritated mucosa: this action is defined as “demulcent” activity. Demulcent indicates a soothing and protective effect on the irritated or inflamed mucosal tissue [9].

The aim of this trial was to evaluate the efficacy and safety of KalobaTUSS**®** compared with the placebo on nocturnal and diurnal acute cough in children.

## Methods

Participants were recruited for this study in the paediatric consulting rooms of the Azienda Ospedaliera Provinciale of Messina in collaboration with the Azienda Ospedaliera Universitaria (AOU) Policlinico “G. Martino” of Messina, Italy. The following inclusion criteria were adopted: children aged 3–6 years with acute cough that had persisted for at least three consecutive days, prompting parents to seek a paediatrician consultation. Exclusion criteria were a cough lasting more than 3 weeks, children with a history of obstructive pulmonary diseases, heart diseases, cystic fibrosis, diabetes, neurological diseases, and immunodeficiencies. After the visit to the paediatrician to exclude asthma or other hyperactive airway diseases as the cause of cough, children suitable for the study according to the inclusion criteria were randomly allocated to one of the two treatment groups (KalobaTUSS**®** or placebo).

Parents signed the informed consent form for children to participate in this trial. Random allocation to the two treatment groups was performed by in a blinded manner by paediatricians who were not aware of the treatment assignment as described below. At admission, subjects were assigned identification numbers and randomly allocated to treatment groups through an external centre for randomization using block randomization with the 1:1 allocation method and blocks of 2 and 4. Allocation concealment was guaranteed by the use of numbered containers. The KalobaTUSS**®** or placebo assignment was sealed in sequentially numbered identical opaque and sealed containers according to the allocation sequence. Containers were opened sequentially only after the child’s name was written on the envelope. Both participants (their parents) and care providers were double-blinded regarding the interventions, and group membership was disclosed after the analysis of the results. Children belonging to the active group received the KalobaTUSS**®** syrup and the second group received a placebo as the syrup formulation. Each of two treatment regimens, KalobaTUSS**®** or placebo, was administered in 4 doses daily in 5 ml per dose for 8 days. The placebo syrup contained 20% fructose and excipients, but it did not contain honey or plant extracts. The appearance, consistency, organoleptic characteristics and viscosity of KalobaTUSS**®** and the placebo were very similar. Therefore, the effect of the KalobaTUSS**®** syrup on the verum group might be associated with the honey and plant extracts.

The study was approved by the Ethics Committee of AOU Policlinico “G. Martino” with protocol number 95/18 on 17 December 2018. The trial was conducted according to the ethical principles of the Declaration of Helsinki and Good Clinical Practice principles were adopted. The size of the sample to enrol in the study was calculated based on an average baseline cough score of 3 points in both groups, and an average change in the cough score of 2 points occurring in the group treated with the study product and 1 point for the placebo group (ClinicalTrial.gov identifier: NCT04073251).

The primary outcome of interest was the change in the night-time cough score before treatment (N0) and nocturnal scores obtained after the first (N1), fourth (N4) and eighth nights of treatment, and the change in the diurnal time cough score before treatment (D0) and scores obtained after the first (D1), fourth (D4) and eighth days of treatment. The secondary outcome was the safety of the syrup when administered to children with acute cough for 8 days, which was evaluated by recording potential adverse reactions to treatment.

Cough was clinically diagnosed and evaluated by paediatricians during the first visit and successively monitored by parents, who completed a daily diary after receiving instructions from the paediatricians. The clinical efficacy of KalobaTUSS**®** was assessed using a validated 6 points Likert scale that was completed by parents and chosen because it is able to establish the severity of cough and to measure the effects of the treatments. The 6 points Likert scale was used to measure the effects of the interventions on all outcomes. The scale ranged from 0 (absence of cough) to 5 (disturbing cough) points corresponding to six increasing degrees of severity for cough at night-time and six other increasing degrees of severity for cough during the day [26]. The syrup was always administered by parents. At the end of the administration period, paediatricians examined the children again and collected the daily diary completed by the parents for each child.

The mean period of coughing before recruitment was calculated to be 3.53 (± SD 0.57) days for the group of children taking KalobaTUSS**®** syrup and 3.52 (± SD 0.58) days for the group treated with the placebo, and the difference between the two groups was not significant (*p* = 0.93624). None of the children monitored for the study required antibiotics or any other pharmacological therapy during the treatment period or during the follow-up period of 1 week after the end of the study.

Adverse events (AEs) were monitored throughout the clinical study. AEs were described as inappropriate medical events that were or were not associated with the procedures or the product.

### Statistical analysis

The statistical analysis of changes in the cough score between the treatment groups was performed using the Mann-Whitney U test. Demographic data are reported as means ± standard deviations (SD), changes produced by treatments are presented as means ± standard errors (SE). Estimated effect sizes (Cohen *d* with Hedges bias correction) with 95% CIs are provided for night-time and day-time cough scores. Evaluations of events (cough) between the two groups are reported as the relative risk and relative risk reduction (RR and RRR, respectively), along with 95% confidence intervals (CI) and *p*-values. A *p*-value less than 0.05 was considered statistically significant (Table 5).

## Results

**Fig. 1:**
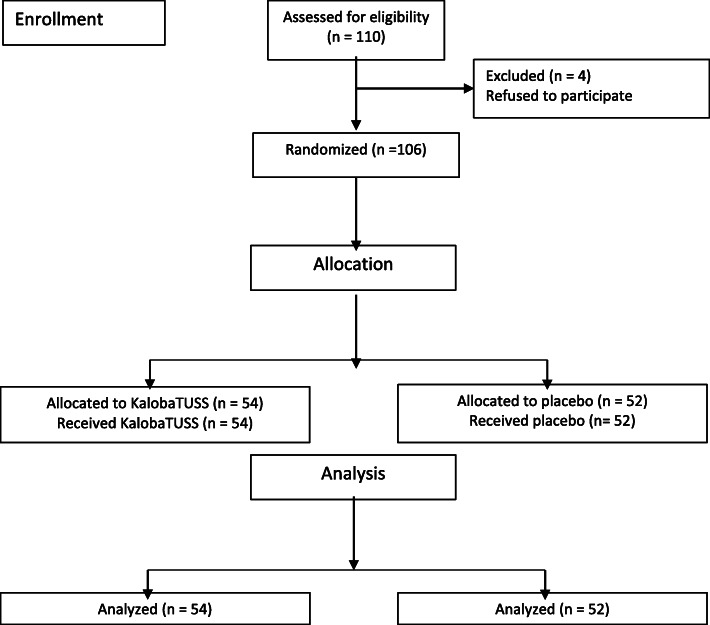
Study Flowchart

One hundred ten (110) children with acute cough were consecutively enrolled and divided into two groups. Enrolment began on 15 February 2019 and was completed on 10 May 2019. Four children, two from each group, left the study before beginning treatment. One hundred six children began and completed the treatment (Fig. 1). Fifty-four (54) children received the KalobaTUSS**®** syrup and another group composed of fifty-two (52) children received the placebo syrup. The median age of children completing the study was 53.02 ± a standard deviation (SD) of 12.02 months (range, 38–70 months; median age 4.3 months), with no significant difference in age between the two treatment groups (*p* = 0.8181). Boys and girls accounted for 50.8 and 49.2%, respectively, of the total sample of enrolled children. Similar percentages of boys and girls were included in the KalobaTUSS**®** and placebo groups (Table 1).

**Table 1:** Baseline characteristics of children enrolled for the study

	KalobaTUSS®	Placebo	Whole sample	Statistics
Children n.	54	52	106	
Mean age (months)	53.2 ± 11.8	52.9 ± 12.3	53.0 ± 12.0	NS
Median age (months)	52	51.5	52	__
Sex (male/female)	28/26	26/26	54/52	NS
Consecutive days of cough before recruitment	3.53 ± 0.57	3.52 ± 0.58	3.59 ± 0.65	NS

Data are expressed as means ± standard deviation. *NS* Not significant

### Night-time cough

**Fig. 2:**
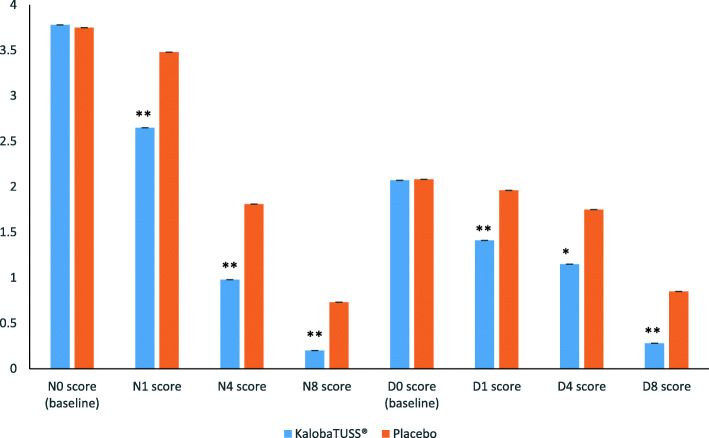
Night-time or Day-time cough score before and after treatment with KalobaTUSS**®** or placebo. * = *p* < 0.05 vs placebo; * = *p* < 0.01 vs placebo

**Fig. 3:**
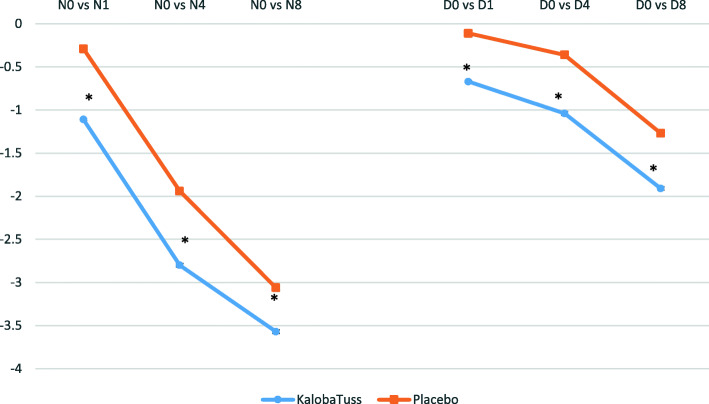
Reduction in night-time and day-time cough scores in children treated with KalobaTUSS**®** or Placebo. * = *p* < 0.01 vs Placebo

Children who received the KalobaTUSS**®** syrup already showed a significant reduction in night-time score after the first day of treatment, 1.11 ± 0.01 (mean ± SE) from N_0_ (last night before treatment) to N_1_ (first night after treatment), compared to a 0.29 ± 0.01 change in the N_0_ to N_1_ score for children receiving the placebo syrup (*p* = 0.00001), thus indicating a quick improvement in night-time cough (Tables 2 and 4 and Fig. 2). A reduction in the scores compared with N_0_ was also observed after four or eight nights of treatment. This effect was observed when comparing the scores obtained after four (N_4_) or eight nights (N_8_) with the score recorded on N_0_ or by comparing the total sum of nocturnal scores after four nights or eight nights of treatment with KalobaTUSS**®** with the night scores of the placebo group. The application of the formulas developed by Hedges and Cohen showed a large effect size after the first night and at the end of treatment compared to the placebo (Table 2). Differences in the reduction of nocturnal cough scores between N_0_ (baseline) and N_1_ (night 1), between N_0_ and night 4 (N_4_) or between N_0_ and night 8 (N_8_), were greater in children treated with KalobaTUSS**®** than in the placebo group: the N_0_ vs N_1_ score was − 1.11 ± 0.01 (SE) for the KalobaTUSS**®** group and - 0.29 ± 0.01 (SE) for the placebo group; the N_0_ vs N_4_ score was − 2.80 ± 0.02 (SE) for the KalobaTUSS**®** group and − 1.94 ± 0.02 (SE) for the placebo group; and the N_0_ vs N_8_ score was − 2.80 ± 0.02 (SE) for the KalobaTUSS**®** group and − 1.94 ± 0.02 (SE) for the placebo group (Table 4 and Fig. 3). Nocturnal cough was no longer present in a larger group of children treated with KalobaTUSS**®** compared to the placebo group when assessed either after 4 days or 8 days of treatment (Table 5).

**Table 2:** Night-time cough score before and after treatment with KalobaTUSS**®** or placebo

Score	KalobaTUSS®	Placebo	Effect size *(Hedges’ g)*	Effect size *(Cohen’s d)*	p
N_0_ score (baseline)	3.78 ± 0.01	3.75 ± 0.02	0.036806	0.036776	0.8493
N_1_ score	2.65 ± 0.02	3.48 ± 0.02	0.760777	0.76118	0.0006
N_4_ score	0.98 ± 0.02	1.81 ± 0.02	0.737756	0.736129	0.0008
N_8_ score	0.20 ± 0.01	0.73 ± 0.02	0.605444	0.602798	0.0075
Total score of first 4 nights	6.46 ± 0.07	9.36 ± 0.08	0.734184	0.733648	0.0003
Total score of all 8 nights	8.20 ± 0.11	13.40 ± 0.11	0.838006	0.83697	0.00001

Data are expressed as means ± standard error. N = night; p was considered significant when < 0.05

### Day-time cough

KalobaTUSS**®** significantly reduced the day-time score after the first day of treatment in the interval from D_0_ (last day before treatment) to D_1_ (first day after treatment). The day-time score from D_0_ to D_1_ was reduced by 0.67 ± 0.01 (mean ± SE) points in the KalobaTUSS**®** group compared to 0.11 ± 0.01 in the placebo group (*p* < 0.00001), thus indicating a quick improvement in the day-time cough as well. The improvement in cough was subsequently revealed by the larger difference in the reduction in the day-time cough score from D_0_ to D_1_ (− 1.11 ± 0.01) in the group of children treated with KalobaTUSS**®** compared to the placebo treatment (− 0.29 ± 0.01) (*p* = 0.00001). A significant difference in the reduction of cough scores was also observed after 4 days of treatment. This effect was observed when we compared the score recorded on the fourth day (D_4_) with the score on D_0_ or by comparing the sum of daily cough scores after 4 days of treatment with KalobaTUSS**®** with the scores recorded at the same time for the placebo group (Tables 3 and 4 and Fig. 2). The effect size of KalobaTUSS**®** on daytime cough was larger than the effect of the study syrup on night-time cough after the first night and at the end of treatment (Table 3). Differences in diurnal cough scores between D_0_ and D_1_ (day 1), D_4_ (day 4) or D_8_ (day 8) were greater in children treated with KalobaTUSS**®** than in the placebo group (Fig. 2). The D_0_ vs D_1_ score was − 0.67 ± 0.01 (SE) for the KalobaTUSS**®** group and − 0.11 ± 0.01 (SE) for the placebo group, the D_0_ vs D_4_ score was − 1.04 ± 0.02 (SE) for the KalobaTUSS**®** group and − 0.36 ± 0.02 (SE) for the placebo group, and the D_0_ vs D_8_ score was − 1.91 ± 0.02 (SE) for the KalobaTUSS**®** group and − 1.27 ± 0.03 (SE) for the placebo group (Table 4 and Fig. 3). Diurnal cough was no longer present in a larger percentage of children treated with KalobaTUSS**®** in comparison to the placebo group, either after 4 days or after 8 days of treatment (Table 5). No AEs were observed throughout the whole duration of the clinical study in the two groups of treatment.

**Table 3:** Day-time cough score before and after treatment with KalobaTUSS**®** or placebo

Score	KalobaTUSS®	Placebo	Effect size *(Hedges’ g)*	Effect size *(Cohen’s d)*	p
D_0_ score (baseline)	2.07 ± 0.01	2.08 ± 0.01	0.013795	0.013793	0.9442
D_1_ score	1.41 ± 0.01	1.96 ± 0.01	0.794567	0.793031	0.0004
D_4_ score	1.15 ± 0.02	1.75 ± 0.02	0.543784	0.542748	0.0139
D_8_ score	0.28 ± 0.01	0.85 ± 0.02	0.682739	0.676823	0.0063
Total score of first 4 nights	5.09 ± 0.05	7.58 ± 0.06	1.041877	1.031956	0.0002
Total score of all 8 nights	7.33 ± 0.11	11.83 ± 0.12	0.766493	0.764947	0.0002

Data are expressed as means ± standard error. D = day; p was considered significant when < 0.05

**Table 4:** Differences in cough scores of the night (N_0_ score; baseline) and the day (D_0_ score; baseline) before enrollment compared with night 1 (N_1_), night 4 (N_4_), night 8 (N_8_), and day 1 (D_1_), day 4 (D_4_), day 8 (D_8_), respectively

	KalobaTUSS®	Placebo	p
N_0_ vs N_1_	- 1.11 ± 0.01	- 0.29 ± 0.01	0.00001
N_0_ vs N_4_	- 2.80 ± 0.02	−1.94 ± 0.02	0.00014
N_0_ vs N_8_	- 3.57 ± 0.02	- 3.06 ± 0.02	0.00758
D_0_ vs D_1_	- 0.67 ± 0.01	- 0.11 ± 0.01	0.00036
D_0_ vs D_4_	- 1.04 ± 0.02	- 0.36 ± 0.02	0.00804
D_0_ vs D_8_	- 1.91 ± 0.02	- 1.27 ± 0.03	0.03940

Data are expressed as means ± standard error. p was considered significant when < 0.05

**Table 5:** Evaluation of events (cough) between the two groups is expressed as Relative Risk and Relative Risk reduction (RR, RRR), 95% confidence intervals (CI) and *p*-values. A *p*-value smaller than 0.05 was considered statistically significant

	KalobaTUSS® (children with cough)	Placebo (children with cough)	*RR (95% CI)*	*RRR (95% CI)*	*p*
Night time cough after 4 days of treatment	34/54	43/52	0.76 (0.6–0.9)	0.24 (0.032–0.40)	0.0256
Night time cough after 8 days of treatment	7/54	22/52	0.306 (0.143–0.65)	0.69 (0.34–0.85)	0.0023
Day time cough after 4 days of treatment	38/54	45/52	0.81 (0.66–0.99)	0.18 (0.0032–0.33)	0.046
Day time cough after 8 days of treatment	8/54	24/52	0.32 (0.16–0.65)	0.68 (0.35–0.84)	0.0016

## Discussion

Cough is frequent in children and its presence is responsible for numerous visits to paediatricians. It represents a separate clinical entity when it occurs in the absence of an organic origin, and its persistence is generally considered a functional disorder [27].

In the present study, we describe a clinical investigation designed to evaluate the potential beneficial effects of the KalobaTUSS**®** syrup containing extracts of *Malva sylvestris*, *Inula helenium*, *Plantago major* and *Helichrysum stoechas*, and acacia honey on acute cough in children. The study product treated cough through a mechanical mode of action: its active substances, honey and herbal extracts, were specifically chosen for their abilities to form a barrier over the irritated mucosa and indirectly reduce the throat inflammation that triggers the cough reflex.

Preclinical data obtained from in vitro experiments (not yet published) showed that the study syrup possesses very good mucoadhesive properties in human oral mucosa cells (static mucoadhesiveness) and is also able to form a mucoadhesive layer (dynamic mucoadhesiveness) that is sufficient to resist the action of the mucosal liquids with which it comes into contact. This peculiarity, namely, the formation of a compact mucosal layer and, in particular a more resistant layer, results in a greater permanence of the product on the mucous membranes and therefore in a greater and prolonged protective effect.

The results of the present clinical trial show a significant reduction in night-time (N) and day-time (D) cough symptoms in children receiving KalobaTUSS**®** syrup, as measured using a specific evaluation scale. Cough score indicated that parents rated KalobaTUSS**®** as better than placebo. Reductions in both night- and day-time scores were rapidly observed after 24 h, as evidenced by the evaluation of the differences between basal night (N_0_ score) and day (D_0_ score) scores and the scores recorded during the next night (N_1_ score) and day (D_1_ score). Moreover, the antitussive effects were maintained after 4 days of treatment, based on the comparison of the scores recorded for children treated with KalobaTUSS**®** or the placebo with basal values or the comparison of the total sum of daily scores obtained by recording the score for each child after 4 days of treatment with KalobaTUSS**®** with the scores of the placebo group.

Scores obtained with the questionnaire completed by parents showed a greater reduction in the severity of night-time cough by treatment with the study syrup than the reduction in the severity of the day-time cough: *P* < 0.01 (= 0.00758) after 8 nights and *P* < 0.05 (= 0.03940) after 8 days. We did not further investigate this result, although diurnal cough is influenced by diurnal changes in respiratory physiology, and changes in environmental triggers may be confounding factors in the determination of cough [28]. Nevertheless, the reduction in night-time cough scores is a very important topic in cough management as a reduction in night-time cough is difficult to obtain, causes considerable discomfort and produces sleep disturbances both in children and their parents.

Although the analysis was not complete, the relief of symptoms by the study syrup was already significant after the first 24 h, thus suggesting an early response to treatment. The early effect was also associated with a quick relief of cough. The fastest relief was observed in a larger percentage of children without nocturnal and diurnal cough in the group treated with the study syrup than in the group of children treated with placebo after both 4 and 8 days of treatment. The safety of the antitussive product has been assessed, with no reports of adverse events. The palatability, colour, door and density of both the study syrup and placebo syrup were also acceptable.

Despite the inherent limitation associated with the indirect collection of data by the children’s parents, this clinical trial suggests acacia honey together with specific fractions derived from *Malva sylvestris*, *Inula helenium*, *Plantago major*, and *Helichrysum stoechas* extracts exert beneficial effects on acute cough in children. The significant improvement observed in the cough score may be attributed to the demulcent and expectorant characteristics of the study product. While antitussive drugs act either on the cough centre of central nervous system that controls coughing or on the peripheral cough receptors located in the trachea, pharynx and branch points of large airways and distal smaller airways, the effects of many natural substances on cough are due to the protective and demulcent activities of mucilage [29].

The ingredients of KalobaTUSS**®** create a protective film on the mucosa, calming cough and protecting the upper respiratory tract. Active components include a large amount of polysaccharides and mucilage that create a film barrier on the oropharyngeal and larynx mucosa through a bioadhesion mechanism of action. The adhesion of these components to the upper respiratory tract mucosa limits the contact with external irritating agents, promotes hydration and limits irritative processes [30].

In addition to the known protective effects of mucilage, and anti-inflammatory and antimicrobial properties of honey, the palatability of honey itself might promote salivation, reducing cough caused by low levels of irritation in the larynx and pharynx [31]. In conclusion, KalobaTUSS**®** is well tolerated and exerts positive effects by reducing the severity and shortening the duration of cough in children.

## Data Availability

The datasets used and/or analysed during the current study available from the corresponding author on reasonable request.

## Abbreviations

AOU: Azienda Ospedaliera Universitaria
AEs: Adverse events
SD: Standard deviation
SE: Standard error
RR: Relative risk
RRR: Relative risk reduction
CI: Confidence intervals
N: Nighttime
D: Day-time
